# Transcriptome analysis highlights nuclear control of chloroplast development in the shoot apex

**DOI:** 10.1038/s41598-018-27305-4

**Published:** 2018-06-11

**Authors:** Vijay Dalal, Shlomi Dagan, Gilgi Friedlander, Elinor Aviv, Ralph Bock, Dana Charuvi, Ziv Reich, Zach Adam

**Affiliations:** 1 The Robert H. Smith Institute of Plant Sciences and Genetics in Agriculture, The Hebrew University of Jerusalem, Rehovot, 76100 Israel; 2 Department of Biomolecular Sciences, Weizmann Institute of Science, Rehovot, 76100 Israel; 3 The Mantoux Bioinformatics institute of the Nancy and Stephen Grand Israel National Center for Personalized Medicine, Weizmann Institute of Science, Rehovot, 76100 Israel; 4 Max Planck Institute of Molecular Plant Physiology, 14476 Potsdam-Golm, Germany; 5 Institute of Plant Sciences, Agricultural Research Organization - Volcani Center, Rishon LeZion, 7505101 Israel

## Abstract

In dicots, the key developmental process by which immature plastids differentiate into photosynthetically competent chloroplasts commences in the shoot apical meristem (SAM), within the shoot apex. Using laser-capture microdissection and single-cell RNA sequencing methodology, we studied the changes in the transcriptome along the chloroplast developmental pathway in the shoot apex of tomato seedlings. The analysis revealed the presence of transcripts for different chloroplast functions already in the stem cell-containing region of the SAM. Thereafter, an en masse up-regulation of genes encoding for various proteins occurs, including chloroplast ribosomal proteins and proteins involved in photosynthesis, photoprotection and detoxification of reactive oxygen species. The results highlight transcriptional events that operate during chloroplast biogenesis, leading to the rapid establishment of photosynthetic competence.

## Introduction

The light-dependent reactions of oxygenic photosynthesis are carried out within extensive networks of flattened vesicles, called thylakoids. The most advanced form of these networks is found in higher-plant chloroplasts, where they form one of the most complex membranous systems in cells^1–6^. Yet, this massive and elaborate system develops essentially from scratch, commencing in undifferentiated plastids termed proplastids, which contain little or no photosynthetic proteins or internal membranes. Bridging the enormous compositional and functional gaps between proplastids and mature chloroplasts requires an array of tightly controlled processes including plastid-nucleus signaling, extensive transcription and translation of nuclear and plastidial genes, massive synthesis of lipids, import of proteins into plastids, insertion of proteins into the thylakoid membranes, assembly of the proteins and incorporated pigments into functional complexes, and differentiation of the lamellar system into its mature, competent form (for some reviews, see^5–12^).

**Figure 1:**
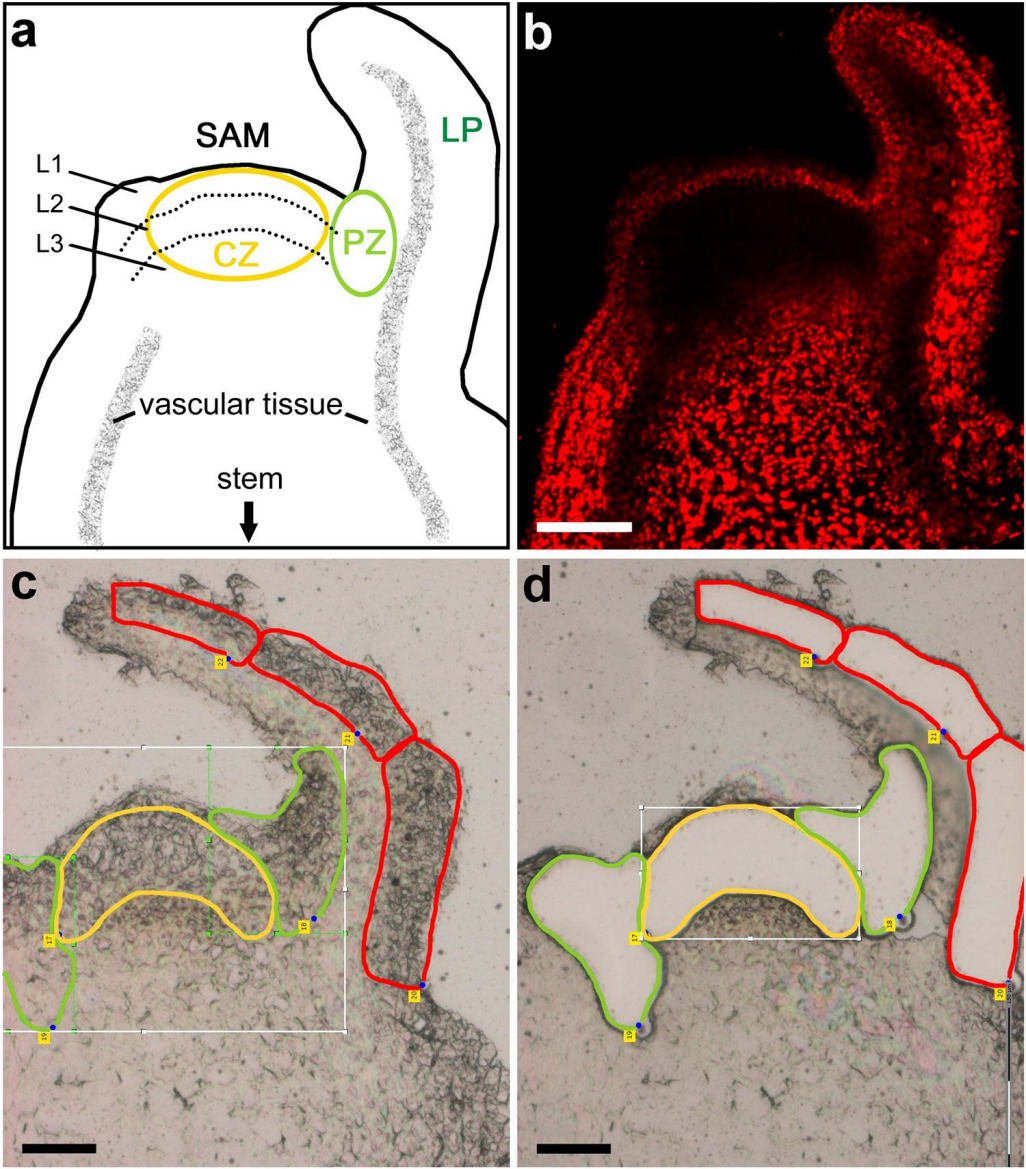
The vegetative shoot apex of tomato. (**a)** Illustration of the vegetative shoot apex, which is comprised of the shoot apical meristem (SAM) and leaf primordia (LP). The central zone (CZ) of the SAM contains stem cells that give rise to all of the aerial parts of the plant. Surrounding the CZ is the peripheral zone (PZ), from which leaf organs are formed. The three clonal layers of the SAM (L1, L2, L3), which generate the different tissues of the leaf, are also marked. **(b)** Chlorophyll fluorescence image of the SAM and young leaf primordium. **(c**,**d)** A typical section of the shoot apex before **(c)** and after **(d)** being subjected to laser capture microdissection, to isolate the chlorophyll-less region of the SAM CZ (yellow outline), the PZ, in which chlorophyll fluorescence becomes visible (green outline), and tissue of the LP which harbors still more developed chloroplasts (red outline). For better visibility, the original software lines were re-traced. Scale bars, 50 μm.

In dicots, which constitute the largest group of flowering plants (angiosperms), the aforementioned processes are initiated in the shoot apical meristem (SAM) and flanking leaf primordia (LP), collectively termed the ‘shoot apex’ (Fig. 1a). The vegetative SAM is comprised of three functionally distinct regions: (1) the central zone (CZ) contains a small number of slowly dividing stem cells that are the source for all of the aerial parts of the plant, (2) the peripheral zone (PZ) surrounds the CZ and generates leaf organs, and (3) the rib zone (RZ), which is located beneath the CZ, supplies cells for the internal tissues of the stem and leaves. The SAM is also divided into three distinguished clonal layers transversely, each of which gives rise to different tissues of the leaf. The outermost layers, L1 and L2, generate the epidermis and the outer mesophyll, respectively, with the latter constituting the main photosynthetic tissue of the leaf. The inner layer, L3, or corpus, which in fact consists of several cell layers, makes up the inner bulk of the SAM and contribute cells toward the outer mesophyll tissue and vasculature.

In a previous study^13^, we characterized the process of chloroplast biogenesis in the shoot apex of Arabidopsis using different microscopic methods. We found that true proplastids reside only in the CZ of the L2 layer and the topmost layer of L3 of the SAM. These proplastids start to develop thylakoid membranes upon arrival of the cells to the SAM’s PZ, within a few cell divisions from the CZ. The membranes continue to expand and differentiate within developing leaf primordia before reaching their mature form. Thylakoid membrane development and the acquisition of photosynthetic competence thus follow a sharp gradient across the shoot apex. Here, we report on the changes in the cellular transcriptomes that take place along this gradient.

## Results and Discussion

Given the small size of the vegetative Arabidopsis SAM (diameter of 50–60 µm), we analyzed the relatively larger SAM of tomato, measuring 150–200 µm in diameter. The pattern of chlorophyll fluorescence in tomato generally resembles that of Arabidopsis^13^, with no fluorescence apparent in the central area of the SAM below the L1 layer, and a sharp increase of fluorescence when moving from the CZ to the PZ, and then to the LP (Fig. 1b). This visual indicator for chloroplast development guided the selection of the desired regions, termed for simplicity as the regions they overlap with – CZ, PZ and LP (Fig. 1). Notably, the pattern of chlorophyll fluorescence exhibited by the different SAM layers and regions, both in Arabidopsis^13^ and tomato, does not correspond to the known gene expression patterns delineating the SAM zones^14^. While studies using fluorescently-labeled markers have provided valuable information on gene expression patterns in defined regions of the SAM^15,16^, these do not reproduce the chloroplast differentiation path. Thus, following the initial events of chloroplast biogenesis necessitated the use of laser-capture microdissection (LCM). A typical section, before and after LCM, is presented in Fig. 1c,d, respectively. As the number of cells in the samples was exceedingly small, only minute amounts of RNA could be extracted, necessitating the use of single-cell RNA sequencing methodology (CEL-Seq^17^). Altogether, a total of ~4,000 unique transcripts were identified, with a mostly similar pattern of gene products’ cellular localizations as the total transcriptome (see below). Notably, almost all of the transcripts identified were present in the three regions analyzed, including in the proplastid-containing region of the SAM (Supplementary Table 1). A list of putative differentially expressed genes (DEGs) was compiled using a threshold ≥ six reads and a cutoff of 1.5-fold expression change. Subsequently, qPCR was performed on 95 genes selected from the list. The final list of 223 DEGs (Supplementary Table 2) included ones that showed a two-tailed FDR ≤0.05, or individual *p*-values ≤ 0.05 for genes whose expression levels were validated by qPCR. The thus compiled list primarily represents genes whose expression level in LP differs from those in CZ (>90%). From CZ to PZ, 39 genes were differentially expressed, 19 of which differed also between CZ and LP. From PZ to LP, 20 genes were differently expressed, the level of 17 of which also differed between CZ and LP.

**Figure 2:**
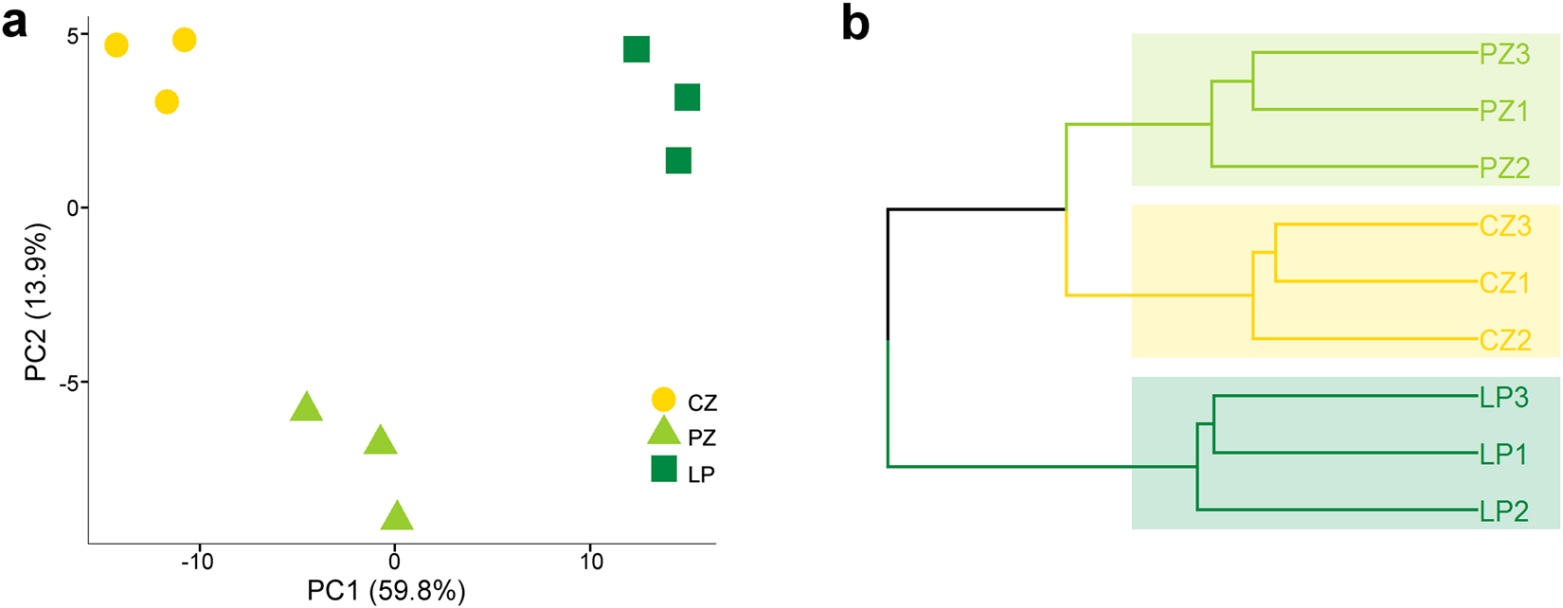
Principal component- and cluster analyses of differentially expressed genes in the shoot apex. (**a**) Principal component analysis (PCA) plot and **(b)** unsupervised hierarchal clustering. CZ, central zone; PZ, peripheral zone; LP, leaf primordia.

**Figure 3:**
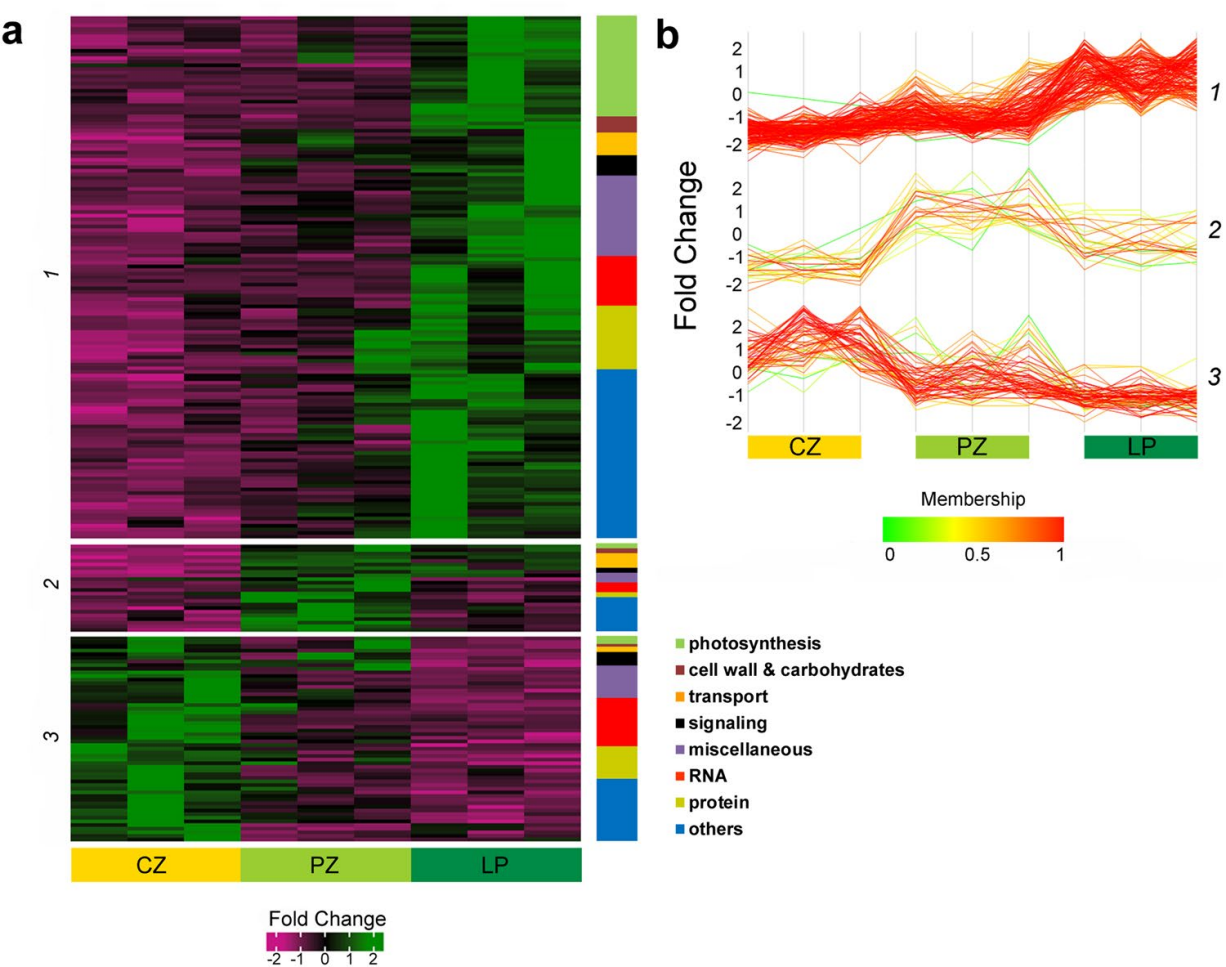
The differentially expressed genes (DEGs) group into three distinct clusters. (**a**) Heat map and the distributions of GO annotations and **(b)** expression profiles of the DEGs belonging to the three clusters. Each gene (**b**) is depicted as a line with the color indicating its similarity to the computed mean profile of the cluster.

Principle component analysis and hierarchical clustering of the DEGs show that the samples taken from the three regions of the shoot apex are well separated from each other (Fig. 2). This is in spite of the PZ being a transition zone between the SAM and leaf organs. Three distinct expression patterns are observed (Fig. 3), with the majority of the genes being upregulated throughout the developmental path. The second largest group is of genes whose expression is downregulated along the gradient. The third consists of genes whose expression first increases, between CZ and PZ, and then decreases upon the transition from PZ to LP. As shown in the bar graph in Fig. 3a, photosynthesis genes are highly represented within the first group and are barely present in the other two.

**Figure 4:**
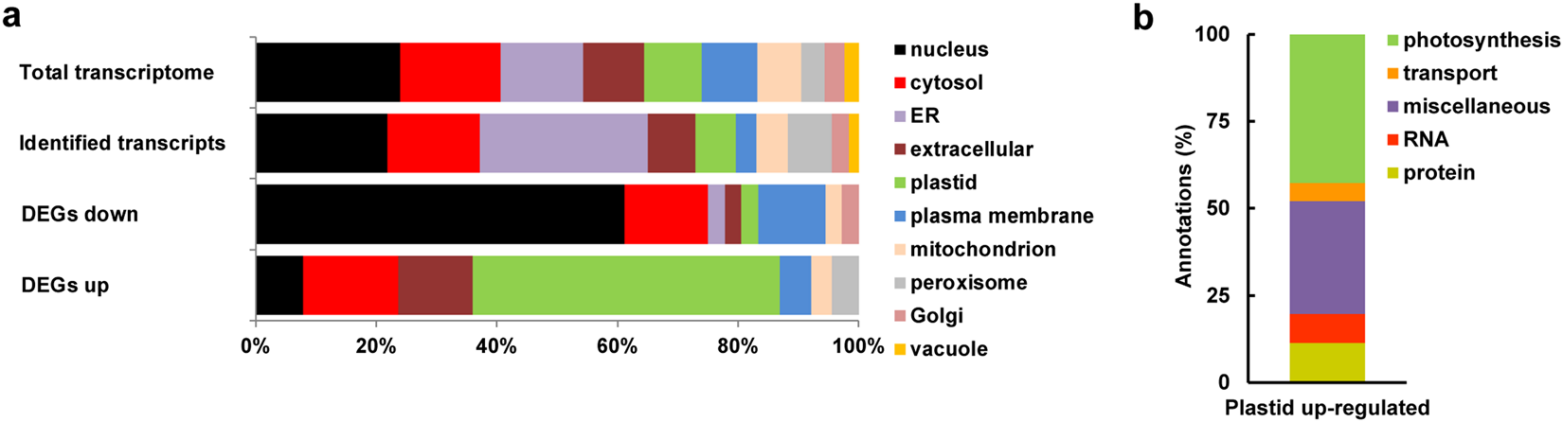
Plastid-bound differentially expressed gene (DEG) products are enriched along the chloroplast developmental gradient. (**a**) Predicted cellular localization (according to SUBA4)^39^ of proteins encoded by genes of the total transcriptome (first bar), identified transcripts (second bar), and down- (third bar) and up-regulated (fourth bar) DEGs. **(b)** Functions of the up-regulated DEGs predicted to be localized to plastids.

The predicted subcellular localizations of the differentially expressed gene products are shown in Fig. 4a (two lower bars). As can be seen, plastids are the most prevalent destination of these products, amounting to 36% of the total. This enrichment, which also pertains to photosynthesis genes, is highly significant (*p* < 0.01, hypergeometric test). This is despite the fact that these transcripts amount to only 7% of the total we identified in the shoot apex (Fig. 4a, second bar from the top). The other major destinations of the DEG products are the nucleus (20%), and the cytosol (15%). The least common cellular compartment of the DEG products is the ER, which constitutes the target of less than 2% of these products. This is albeit being the predicted site for almost 30% of the proteins encoded by the transcripts identified in the shoot apex. The fact that along the CZ-PZ-LP developmental path almost no changes occur in the expression of genes encoding for ER-targeted proteins may have two explanations. First, it may be that the expression of the relevant genes is already high at the central zone and is maintained as such in the peripheral zone and leaf primordia, perhaps reflecting processes initiated earlier during embryogenesis. Alternatively, low expression levels of ER (and Golgi proteins, see Fig. 4a, two lower bars) may be maintained to reduce secretory load, perhaps as a systemic effort to funnel available resources to thylakoid membrane formation and differentiation and other processes associated with plastid ontogeny. As can be expected, a large fraction (about half) of the plastid-targeted proteins encoded by the up-regulated DEGs are related to photosynthesis (Fig. 4b). Other up-regulated DEG products localized to plastids are involved in RNA and protein synthesis and processing, essential for the construction of the photosynthetic apparatus and its auxiliary components.

**Figure 5:**
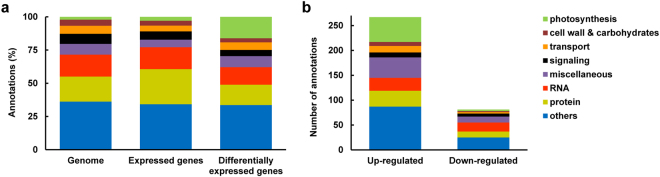
Plastid differentiation in the shoot apex is accompanied by marked increase in the expression of photosynthesis-related genes. (**a**) Distribution of annotations of whole genome (left bar), expressed genes (middle bar), and differentially expressed genes (DEGs, right bar). (**b**) Number of up- and down-regulated DEGs and their functional assignments.

Examination of the DEGs operating along the chloroplast developmental path reveals that the relative proportions of the different functional groups to which they belong generally resemble those of the tomato genome and the expressed genes identified (Fig. 5a). The only notable exception are genes encoding photosynthesis-related proteins, which amount to 17% of the DEGs, as opposed to only 2–3% in the genome and the expressed genes. This highlights the allocation of a significant fraction of the cellular resources toward the build-up the photosynthetic machinery.

Overall, the transcripts of 54 nuclear genes encoding chloroplast-targeted proteins were upregulated during the transition (Table 1). These include transcripts encoding for proteins of the chloroplast 30S and 50S ribosomal subunits, constituents of photosystems I and II (PSI and PSII) and their peripheral antenna complements, ATP synthase and NADH dehydrogenase, and ferredoxin and its cognate oxidoreductase. Others were transcripts encoding enzymes of the Calvin-Benson cycle, including the small subunit of ribulose-1,5-bisphosphate carboxylase/oxygenase (Rubisco), glyceraldehyde 3-phosphate dehydrogenase, fructose-bisphosphate aldolases, fructose-1,6-bisphophatase, phosphoribulokinase, and Rubisco activase. Notably, almost all of the above transcripts were already present in the CZ, indicative of an early acquisition of photosynthetic capacity. This is in contrast to maize, where very few photosynthesis-related genes were found to be expressed in the SAM and early-stage leaf primordia^18^. The expression of constituents of the machineries that drive the light-dependent and -independent reactions of photosynthesis was accompanied by the establishment of photoprotective and reactive oxygen species (ROS) detoxification capabilities, along with other oxidative stress resistance mechanisms (Table 1). Such capabilities are especially essential during biogenesis of the photosynthetic apparatus, when chlorophyll and other pigments are synthesized. During this time, light absorption by these pigments can lead to rapid generation of ROS and thus to damage. Non-chloroplastic upregulated DEGs mostly belonged to several major functional groups. These included genes encoding for proteins involved in biotic and abiotic stresses, including oxidative stress, cell wall and carbohydrate metabolism, protein catabolism, and water homeostasis (Supplementary Table 2). The number of downregulated DEGs identified was significantly lower and generally not enriched in particular functional groups, with the exception of several transcription factors (TFs), as described below.

**Table 1:** Expression of genes encoding chloroplast-targeted proteins^†^.

Index	Gene	Protein	Function	Log_2_ Fold change
**Ribosomal proteins**				
5	01g057830	RPS1	30S ribosomal protein, RNA binding	2.85
103	05g009570	PSRP-3	30S ribosomal protein	2.46
189	10g081100		50S ribosomal protein	2.73
202	11g066410	RPL9	50S structural ribosomal protein	4.38
203	11g068820	RPL27	50S structural ribosomal protein	3.57
223	12g100160	RPL6	50S ribosomal protein, RNA binding	2.79
**PSII**				
21	01g105030	LHCB6	chlorophyll a/b binding	2.26
29	02g065400	PSBO2	oxygen evolution	2.03
36	02g070940	LHCB1.4	chlorophyll a/b binding	2.86
44	02g079950	PSBQ	oxygen evolution	2.06
57	02g090030	PSBO	oxygen evolution	1.93
61	03g005760	LHCB1.3	chlorophyll a/b binding	1.15
62	03g005770	LHCB1.3	chlorophyll a/b binding	2.27
105	05g025600	PSBX	reaction center protein	2.61
118	06g063370	LHCB5	chlorophyll a/b binding	1.26
141	07g044860	PSBP	oxygen evolution	2.23
142	07g047850	LHCB2.4	chlorophyll a/b binding	2.06
152	07g063600	LHCB3	chlorophyll a/b binding	2.46
154	07g066310	PSBR	oxygen evolution	1.82
168	09g014520	LHCB4.2	chlorophyll a/b binding	1.41
188	10g077120	PSBY	core complex protein	2.63
221	12g099650	PSBT	unknown	2.90
**PSI**				
33	02g069450	PSAF	PC-docking	3.39
72	03g115900	LHCA4	chlorophyll a/b binding	2.46
76	03g120640	PSAH	LHC-docking	2.11
127	06g074200	PSAO	balancing excitation energy	1.81
155	08g006930	PSAK	peripheral LHC organization	1.80
156	08g013670	PSAN	PC-docking	1.36
170	09g063130	PSAE	Fd-docking	1.28
179	10g006230	LHCA2	chlorophyll a/b binding	2.13
181	10g007690	LHCA3	chlorophyll a/b binding	2.36
210	12g011280	LHCA3	chlorophyll a/b binding	3.69
213	12g044280	PSAH	LHC-docking	2.36
**Carbon metabolism**				
24	01g110360	FBA2	fructose-bisphosphate aldolase	3.57
50	02g084440	FBA2	fructose-bisphosphate aldolase	3.06
51	02g085950	RBCS	CO_2_ fixation	1.89
66	03g034220	RBCS	CO_2_ fixation	2.01
83	04g009030	GAPA1	glyceraldehyde-3-p dehydrogenase	2.13
97	04g082630	GAPB	glyceraldehyde-3-p dehydrogenase	3.67
109	05g052600	FBPASE	fructose-1,6-bisphophatase	2.50
160	08g076220	PRK	phosphoribulokinase	2.08
193	10g086580	RCA	rubisco activase	2.48
196	11g007990		malate dehydrogenase	2.83
**Miscellaneous**				
**8**	01g080280	GS2	glutamine synthase	3.12
**31**	02g066920	CRR7	NDH assembly	3.03
49	02g083810	FNR2	ferredoxin-NADP^+^ reductase	1.39
74	03g118410	ACP4	acyl carrier, lipid synthesis	1.80
96	04g081970	CDSP32	thioredoxin, redox regulation	4.36
106	05g032660	NOL	chl b reductase	2.48
117	06g060340	PSBS	non-photochemical quenching	1.74
183	10g044520	FED	ferredoxin, electron transfer	0.91
191	10g083650	PRXIIE	peroxiredoxin, ROS detoxification	1.93
216	12g056830	ATPD	ATP synthesis	3.05
**Unknown**				
26	02g049070			2.09
122	06g068670			2.14
131	06g076790			2.11

^**†**^Fold changes (*p* < 0.05) indicated in the table are between the leaf primordia and the central zone of the shoot apical meristem.

Table 2 lists TFs and development-related proteins whose expression was up- or down-regulated along the chloroplast developmental pathway. Amongst the former are two *YABBY* genes, *YABBY*1 and *YABBY*2, encoding key TFs involved in abaxial cell fate determination^19^. An opposite behavior is observed for *STM*, a key TF required for SAM formation, which is down-regulated in leaf primordia^20^. *CUC2*, a TF that determines the border of the CZ^21^ is also down-regulated outside this region. These trends faithfully capture known expression patterns of key SAM transcriptional regulators along the shoot apex. The expression of other TFs and developmental regulators displayed changes that are also consistent with processes known to occur in the SAM. *CRK*, a gene encoding an adapter protein involved, amongst other things, in the establishment of cell polarity^22^, was up-regulated along the developmental gradient (Table 2). The expression of the auxin-efflux transporter MDR1, also essential for cell polarity^23^, likewise increased along the gradient. Transcripts of *FD*, *SPT* and *SVP*, all encoding TFs involved in flowering^24^, were identified in the CZ, and down-regulated outside of it, in accordance with the vegetative state of the SAMs we analyzed. Also down-regulated was the gibberellic acid receptor GID1. This gene was the only one whose expression changed exclusively between the peripheral and central zones of the SAM, consistent with the high rate of cell division in this region. Overall, the expression patterns observed for the aforementioned TFs and regulators matches those expected for ontogenetic processes related to leaf growth and differentiation during the vegetative phase.

**Table 2:** Expression of transcription factors and development-related genes operating in the shoot apex^†^.

Index	Gene	Protein	Function	Log_2_ Fold change
11	01g090450	CRK	actin polymerization	4.28
12	01g091010	YABBY1	abaxial cell fate determination (TF)	1.94
14	01g098390	GID1C	GA receptor	−3.15
27	02g061990	FD	transition to flowering (TF)	−3.10
46	02g081120	STM	SAM formation (TF)	−4.24
48	02g083520	FD	transition to flowering (TF)	−1.76
54	02g087870	MDR1	ABC transporter, auxin efflux	3.50
59	02g093280	SPT	floral organogenesis (TF)	−4.97
78	03g123430	AP2	ethylene response (TF)	−2.31
93	04g076280	SVP	flowering (TF)	−1.86
151	07g062840	CUC2	SAM formation (TF)	−4.64
162	08g079100	YABBY3	abaxial cell fate determination (TF)	2.73

^**†**^Fold changes (*p* < 0.05) indicated in the table are between the leaf primordia and the central zone of the shoot apical meristem (SAM), aside from index no. 14, which is between the peripheral and the central zones. TF, transcription factor.

**Figure 6:**
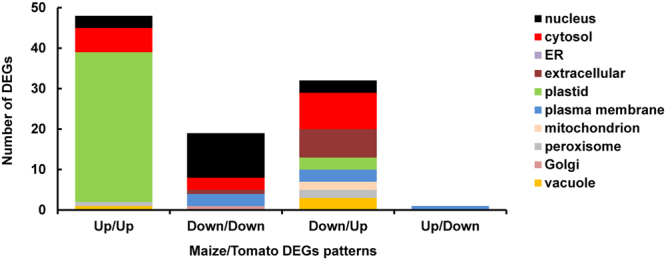
Chloroplast development in tomato is distinct from that in monocots (maize). Cellular localization of up- and down-regulated DEGs across the chloroplast developmental gradients in tomato and maize^11^. Of the 223 DEGs we identified in the tomato shoot apex, about 70 showed a similar behavior in maize leaves (see Supplementary Table 2), the majority of which were up-regulated and encode for plastidial proteins.

Leaves of monocotyledon plants have been widely used as a model for chloroplast development due to their relatively simple architecture and the presence of a linear developmental gradient from the base of the leaf, where proplastids are found, to its tip, possessing the most mature chloroplasts^25–28^. We thus sought to compare our SAM transcriptomic data to those reported for maize leaves^27^. In the latter study, it was found that genes that were upregulated along the developmental gradient were mostly related to protein translation, tetrapyrrole and carotenoid biosynthesis, plastid targeting, photosynthesis, Calvin cycle, redox regulation, very similar to the upregulated DEGs identified in our study (Fig. 6). Of these, the products of 38 genes were chloroplast-targeted. Down-regulated genes observed in both species were related to chromatin structure, DNA replication, cell cycle, signaling and cell wall biosynthesis. Over 30 DEGs that were down-regulated in maize were up-regulated along the developmental path in tomato (Fig. 6). Inspection of these genes does not offer any obvious explanation for the opposite behavior, which might result from differences in species and/or in carbon fixation. This may also hold for other differences observed between the two plants.

The transcriptome analysis performed here revealed that expression of nuclear genes encoding chloroplast-targeted proteins occurs already in proplastid-containing stem cells of the SAM’s CZ, and increases in cells of the PZ and the LP. In this respect, the transition from cell proliferation to cell expansion, highlighted in a previous study^28^ as a key stage in which photosynthetic genes are strongly up-regulated, represents only the continuation of a gene expression gradient that is already established in the SAM. The increasing expression of chloroplast and photosynthesis-related genes, which correlates with thylakoid proliferation and chloroplast development, starts in the SAM and continues all the way through to leaf maturation, and thus, is not limited to specific stages in shoot morphogenesis. This trend is evident not only for chloroplast-related genes, but also for at least one TF controlling their expression, WRKY40^29,30^, which by itself is also found to be up-regulated in our dataset.

The parallels between leaf morphogenesis and chloroplast development, beginning already in the SAM, raise the possibility that the two processes are intimately connected. This is also suggested by the similar expression patterns of chloroplast-related genes and genes related to leaf development (Tables 1 and 2). However, tomato *leafless* (*lfs*) mutants possess a naked shoot apex that is green^31^. Furthermore, some albino mutants that have only proplastids, nonetheless develop normal-looking leaves^32,33^. It therefore seems that the two processes are independent of each other.

Finally, our data show that there is no sequential order of expression of chloroplast-targeted gene products along the chloroplast developmental pathway. As shown in Table 1, genes encoding chloroplast ribosomal proteins, photosynthesis proteins, enzymes related to carbon fixation as well as proteins involved in photoprotection and ROS detoxification are all concurrently expressed. Expression of genes encoding chloroplast proteins thus appears to proceed en masse, beginning already in the CZ of the SAM and progressively increasing towards the flanking primordial leaves. It is quite remarkable that apart from the extensive up-regulation of genes related to chloroplast functions, there appear to be no major changes in the expression of genes related to other organelles and cellular compartments. This is albeit the fact that the developmental gradient encompasses cells belonging to different clones, regions and organs. Future work should aim at increased coverage and spatial resolution using single-cell approaches^34,35^, that potentially can unravel more subtle expression patterns.

## Methods

### Plant material

Tomato (M82 SP^+^) seedlings were grown aseptically in magenta boxes containing half-strength Nitsch medium, under 11 h light/13 h darkness, at 200 μmol photons·m^−2^·s^−1^ and 22 °C. 12–14 day-old seedlings, possessing 5–6 true leaves, were utilized for the experiments.

### Confocal microscopy

A solution of phosphate-buffered saline (PBS) containing 34.7% albumin was poured into 10 × 10 × 5 mm cryo-molds (Sakura Finetek). Albumin cross-linking was achieved by mixing glutaraldehyde (GA, final concentration 1.75%) into the solution, resulting in a hardened block within less than a minute. A ~2-mm-long top part of the seedling containing the shoot apex was placed on top of the block followed by another, identical, round of buffer and GA mix to embed the seedling into the block (the high GA/albumin ratio, utilized to achieve rapid cross-linking of albumin, excludes effective diffusion of GA into the seedlings). The embedded seedlings were then cut to 70-µm-thick slices, using an oscillating tissue slicer (OTS-4000, EMS, USA). All steps were carried out at 4 °C. Imaging was carried out as described^13^.

### Laser capture microdissection (LCM) of SAM microdomains

The top part of the seedling containing the shoot apex was embedded into TissueTek^@^ OCT (Sakura Finetek) medium inside of 10 × 10 × 5 cm cryo-molds. Cryo-blocks were prepared by freezing the SAM-containing cryo-molds in liquid nitrogen and storing them at −80 °C until use. The cryo-blocks were cryo-sectioned into 10-μm-thick slices with a cryostat (Leica CM3050 S) using low-profile blades (MONARCH, Sturkey, US). 12 to 15 such sections were obtained from a single SAM, and collected on 0.1-mm polyethylene terephthalate (PET) or 1.0-mm polyethylene naphthalate (PEN) membrane slides. SAM regions possessing plastids at different developmental stages were selected based on their chlorophyll fluorescence, and LCM was performed on adjacent sequential sections. The sections were then immediately fixed in 70% ethanol, washed and dehydrated by an ethanol series (70, 96 and 100%), dried in air, placed in air-sealed Falcon tubes and stored at −80 °C until use. Cells containing plastids at three progressive developmental stages were laser-microdissected from (a) the chlorophyll-less CZ, (b) the PZ, and (c) the abaxial side of a 150–350-μm-long LP. Laser microdissection was carried out using a PALM MicroBeam LCM system (Carl Zeiss). The cut regions were catapulted into 500-μl adhesive caps (Agilent) containing 35 μl of RLT lysis buffer from the RNeasy Micro RNA isolation kit (Qiagen). Samples were maintained at room temperature for 30 min then stored at −80 °C. RNA quality was assessed with Bioanalyzer 2100 (BA) using a BA-PICO 6000 Chip (Agilent Technologies). Altogether, three biological replicates, each containing three pooled sections, were obtained and subsequently analyzed for each region along the developmental gradient.

### cDNA synthesis, library preparation, RNA sequencing, read alignment, clustering and differential expression analysis

Low input RNA libraries for sequencing were prepared using the CEL-Seq method^17^. Single cDNA library containing all samples was sequenced on a single lane at the Technion Genome Center (Technion – Israel Institute of Technology) with HiSeq2500 (Illumina) using paired-end sequencing. An 18-bp fragment, Read1, was generated for reading barcode and UMI. At the other end, a 36-bp fragment, Read2, was generated for insert cDNA. The quality of sequences was evaluated using FASTQC v0.10.1. Mapping was performed with Bowtie2 v2.1.0 in “Local mode”. Counting of reads to genes was performed with a script based on the HTSeq-counts (provided as part of the CEL-Seq pipeline). Tomato reference genome (SL 2.5) and annotations from Solgenomics, with the addition of the chloroplast genome, were used for counting reads per gene. Differential expression analysis was performed using the R-based software ‘DESeq2’ v1.4.0. Differentially expressed genes were those having ≥6 reads in at least one of the tested zones (CZ, PZ or LP), as well as a fold change (increase or decrease) of ≥1.5 in at least one of the three pair-wise comparisons. Additional criteria were either two-tailed FDR ≤0.05, or individual *p*-value ≤0.05, validated by subsequent qPCR analysis, as detailed below. The differentially expressed signals were log_2_-transformed and normalized by *Z*-score transformation before PCA and cluster analyses. Unsupervised clustering was performed using Euclidean distance metric and average-linkage agglomeration method. The heat map was based on K-means clustering of the genes, using Pearson correlation coefficient as a distance metric. The optimal number of clusters in the heat map was computed using the gap statistic method and included 1000 Monte Carlo iterations^36,37^. The RNA-Seq data have been deposited in NCBI’s Gene Expression Omnibus^38^ and are accessible through GEO Series accession number GSE102070 (https://www.ncbi.nlm.nih.gov/geo/query/acc.cgi?acc=GSE102070).

### Real time PCR (qPCR)

RNA isolation and amplification (for two rounds) were performed as described^3^, except that the primers used were from the MessageAmp II aRNA Amplification Kit (Invitrogene). Three biological replicates were used for each developmental stage, derived from 5–6 SAMs each. cDNA synthesis was carried out using 20 ng of the aRNA. qPCR was performed using the BioMark™ HD System (Fluidigm). Data was analyzed with the Fluidigm Real-Time PCR Analysis software, using the Linear (Derivative) Baseline Correction and the Auto Ct Threshold Method. Differential expression was determined using two-tailed Student’s t-test (p ≤ 0.05).

## Electronic supplementary material

Dataset 1

Dataset 2
